# Improving the Efficiency and Stability of Perovskite Solar Cells by Refining the Perovskite-Electron Transport Layer Interface and Shielding the Absorber from UV Effects

**DOI:** 10.1021/acsami.4c03329

**Published:** 2024-05-27

**Authors:** Salah AL-Shujaa, Peng Zhao, Dingqian He, Basheer Al-Anesi, Yaqing Feng, Jianxing Xia, Bao Zhang, Yi Zhang

**Affiliations:** † School of Chemical Engineering and Technology, Tianjin University, Tianjin 300350, China; ‡ Institute of Molecular Plus, Tianjin University, Tianjin 300072, China; § Haihe Laboratory of Sustainable Chemical Transformations, 300192 Tianjin, China; ∥ Faculty of Engineering and Natural Sciences, Tampere University, Tampere 33014, Finland

**Keywords:** perovskite solar cells, high efficiency and stability, electron transport layer passivation, UV shielding, chemical bath deposition

## Abstract

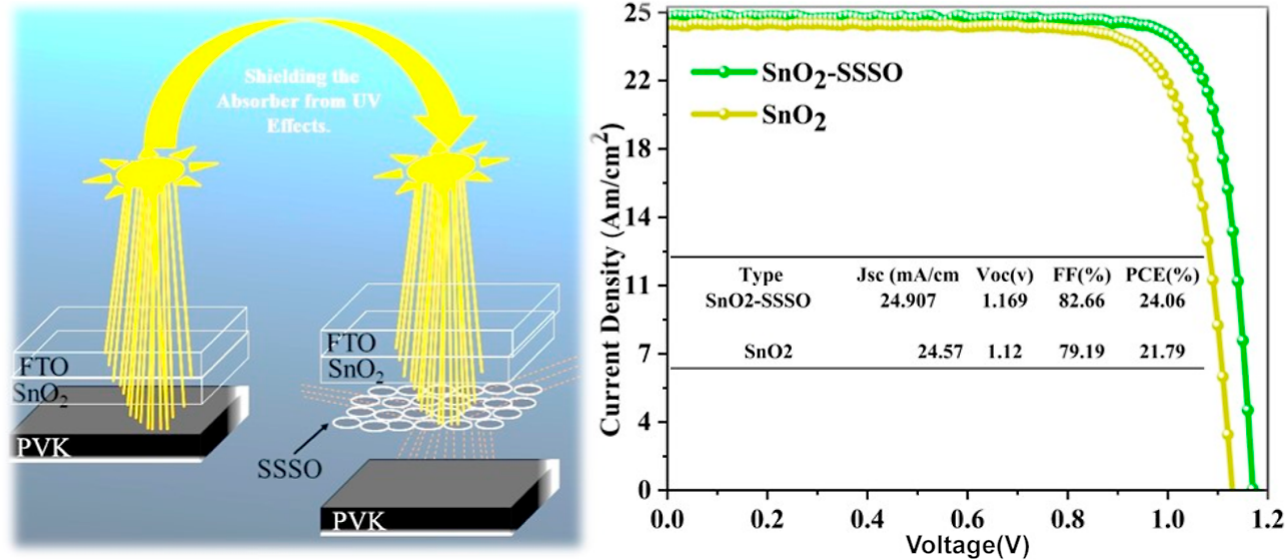

This study aims to enhance the performance of perovskite solar cells (PSCs) by optimizing the interface between the perovskite and electron transport layers (ETLs). Additionally, we plan to protect the absorber layer from ultraviolet (UV) degradation using a ternary oxide system comprising SnO_2_, strontium stannate (SrSnO_3_), and strontium oxide (SrO). In this structure, the SnO_2_ layer functions as an electron transport layer, SrSnO_3_ acts as a layer for UV filtration, and SrO is employed to passivate the interface. SrSnO_3_ is characterized by its chemical stability, electrical conductivity, extensive wide band gap energy, and efficient absorption of UV radiation, all of which significantly enhance the photostability of PSCs against UV radiation. Furthermore, incorporating SrSnO_3_ into the ETL improves its electronic properties, potentially raising the energy level and improving alignment, thereby enhancing the electron transfer from the perovskite layer to the external circuit. Integrating SrO at the interface between the ETL and perovskite layer reduces interface defects, thereby reducing charge recombination and improving electron transfer. This improvement results in higher solar cell efficiency, reduced hysteresis, and extended device longevity. The benefits of this method are evident in the observed improvements: a noticeable increase in open-circuit voltage (*V*_oc_) from 1.12 to 1.16 V, an enhancement in the fill factor from 79.4 to 82.66%, a rise in the short-circuit current density (*J*_sc_) from 24.5 to 24.9 mA/cm^2^ and notably, a marked improvement in the power conversion efficiency (PCE) of PSCs, from 21.79 to 24.06%. Notably, the treated PSCs showed only a slight decline in PCE, reducing from 24.15 to 22.50% over nearly 2000 h. In contrast, untreated SnO_2_ perovskite devices experienced a greater decline, with efficiency decreasing from 21.79 to 17.83% in just 580 h.

## Introduction

Organic–inorganic hybrid PSCs provide a diverse range of beneficial attributes for photovoltaic applications.^1^ These features encompass high absorption coefficients, excellent carrier mobility, extended charge carrier diffusion lengths, cost-effectiveness, and great development potential.^2^ The significant improvements in the efficiency of these solar cells, now at 26.1%,^3^ make them excellent candidates as viable substitutes or rivals for conventional silicon solar cells.^4^ Although this initial challenge has been overcome, they are still unable to meet market demands due to various obstacles: limited operational life,^5^ issues related to stability and degradation,^6^ manufacturing challenges,^7^ cost reduction,^8^ and their performance in outdoor environments.^6^ Thus, to mitigate the impact of these challenges, it is crucial to comprehensively understand and address the issues related to stability and degradation. PSCs are susceptible to a variety of internal and external factors that compromise their stability and contribute to degradation.^9−12^ Internal influence factors pertain to aspects within the perovskite material affecting its performance, including composition, ion movement, bond strength among components, and inherent stability.^13,14^ Conversely, external factors arise from environmental influences and include moisture, temperature, ultraviolet (UV) light, and interface materials.^9−12,15,16^ Researchers have made significant progress in mitigating the impact of external variables.^14,17^ Notably, strategies, such as careful substrate selection^18^ and encapsulation techniques,^19−21^ have been successful in shielding PSCs from external influences. However, thoroughly investigating the effects of UV radiation on PSCs poses a considerable challenge, necessitating further dedicated research and focus.^22,23^

The primary vulnerability of PSCs to UV radiation stems from the high susceptibility of electron-transfer layer (ETL) to photochemical reactions, particularly in the presence of metal oxides.^24^ In addition, UV radiation causes the perovskite to deteriorate into CH_3_NH_2_ and HI. This results in reduced light absorption and conversion efficiency.^25,26^ Consequently, the longevity and diffusion of charge carriers is diminished.^27^ This phenomenon has been confirmed by Song et al., who used mass spectrometry to elucidate the photodecomposition process,^28^ and by Tang et al., who employed X-ray in situ diffraction methods.^29^ To mitigate the detrimental effects of UV radiation on perovskite layers, designers can incorporate a UV-blocking layer into the device’s design. This layer limits the amount of UV radiation that reaches the perovskite layer. UV-blocking layers fall into two main categories: organic and inorganic UV filters. Organic UV filters, which often feature aromatic rings, are composed of carbon-based substances.^30−32^ Contrastingly, inorganic UV filters, composed of metal oxides, absorb or reflect UV radiation, thereby shielding the sensitive perovskite layer from penetration. Moreover, these metal oxides provide long-lasting UV protection, a benefit stemming from their compatibility with the photon energy range of 3.0–3.4 eV.^25,26^ In our preliminary study, we applied CeO_2_ as a passivation layer to absorb UV light, resulting in an increase in PCE to 22.71%, surpassing the 20.7% achieved with SnO_2_ PSCs. After 1700 h of storage, the stability of SnO_2_–CeO_2_ PSCs decreased from 22 to 19%, whereas pure SnO_2_ PSCs experienced a decline from 20 to 16% under identical conditions.^33^

The objective of this study is to enhance the performance of PSCs. This will be achieved by addressing the imperfections at the interface between the perovskite layer and the electronic transport layer and by safeguarding the absorbing layer against the detrimental impact of UV radiation. The accomplishment was realized through the deposition of SrSnO_3_–SrO (SSSO) onto the surface of the SnO_2_ ETL, achieving the SnO_2_–SrSnO_3_–SrO hybrid (SnO_2_–SSSO). The SnO_2_ layer functions as the ETL, strontium stannate (SrSnO_3_) acts as the UV-filtering layer, and strontium oxide (SrO) serves to passivate the interface. SrSnO_3_ is characterized by its chemical stability, electrical conductivity, wide band gap energy, and effective ability to absorb UV radiation. These properties enhance their ability to protect PSCs from environmental elements and UV radiation.^34−36^ In addition, doping SrSnO_3_ into the ETL builds upon its electronic capabilities.^37^ This may enhance the energy level and improve the alignment, leading to higher efficiency in injecting electrons from the perovskite layer to the external circuit.^38−40^ SrO in PSCs serves to mitigate defects at the interface between the perovskite and electron transport layers, decreasing charge recombination and enhancing electron transfer. These improvements result in a higher solar cell efficiency, reduced hysteresis, and enhanced device stability and reliability.^41−44^ Consequently, the incorporation of SnO_2_ as an ETL, SrO passivation, and SrSnO_3_ for UV filtering in perovskite solar cells has led to significant performance improvements. PCE increased by 24.06% (up from 21.79%), open-circuit voltage (*V*_oc_) from 1.12 to 1.16 V, and fill factor (FF) from 79.19 to 82.66%, alongside improved stability, reduced hysteresis, and extended operating life.

## Experimental Section

### Materials

Essential chemical reagents, including thioglycolic acid (TGA, 98%), bis(trifluoroethane) sulfonimide lithium salt (Li-TFSI), 4-*tert*-butylpyridine (tBP), acetonitrile (ACN), dimethyl sulfoxide (DMSO), chlorobenzene (CB), and tris(2-(1*H*-pyrazol-1-yl)-4-tertbutylpyridine)-cobalt(III)Tris(bis(trifluoromethyl sulfonyl)imide) (FK209) were procured from Sigma-Aldrich for the experimental procedures. *N*,*N*-Dimethylformamide (DMF) was also obtained from Sigma-Aldrich. Methylammonium chloride (MACl), lead bromide (PbBr_2_), lead iodide (PbI_2_, 99.99%), and 2,2′,7,7′-tetrakis(*N*,*N*-di-4-methoxyphenylamine)-9,9′-spirobifluorene (spiro-OMeTAD) were acquired from Xi’an Polymer Light Technology. Additionally, Xi’an Polymer Light Technology supplied the lead iodide (PbI_2_, 99.99%) and the 2,2′,7,7′-tetrakis(*N*,*N*-di-4-methoxyphenylamine)-9,9′-spirobifluorene(spiro-OMeTAD). Formamidinium iodide (FAI) was obtained from Greatcell Solar Materials in Australia.

### Device Fabrication

#### Preparation of Perovskite and HTL Solution

The perovskite solution was created by mixing 634.7 mg of PbI_2_, 216 mg of FAI, 30 mg of MACl, and 5.6 mg of MAPbBr_3_ in a DMF/DMSO solution with a ratio of 8:1. Additionally, the hole-transporting layer (HTL) solution was prepared by combining 50 mg of Spiro-OMeTAD, 19.5 μL of tBP, 5 μL of Co(III) TFSI solution (0.25 M in acetonitrile), 11.5 μL of Li-TFSI solution (1.8 M in acetonitrile), and 547 μL of chlorobenzene.

#### Deposition of the Perovskite Layer and HTL

First, 50 μL of perovskite solution was deposited onto the FTO/SnO_2_ substrate using a two-stage spin-coating process: initially at 1000 rpm for 10 s, followed by 5000 rpm for 25 s, each stage employing a ramp of 2000 rpm. Second, just before the final 10 s of this process, 150 μL of CB solution was dropped onto the substrate as an antisolvent. Next, the FTO/SnO_2_/perovskite substrate was annealed at 100 °C for 60 min. Finally, the HTL was applied by dispensing 70 μL of the HTL solution onto the perovskite substrate, followed by spin-coating at 4000 rpm for 20 s, with a ramp-up speed of 2000 rpm/s.

#### Back Electrode (Ag)

In the final step, a layer of Ag, 100 nm thick, was deposited on the film to serve as the back electrode. This process was conducted under a high vacuum (less than 5 × 10^4^ Pa), maintaining an evaporation rate of 1 Å/s. The effective area of the device, measuring 0.09 cm^2^, was precisely defined using a mask throughout the evaporation process.

## Results and Discussion

**Figure 1:**
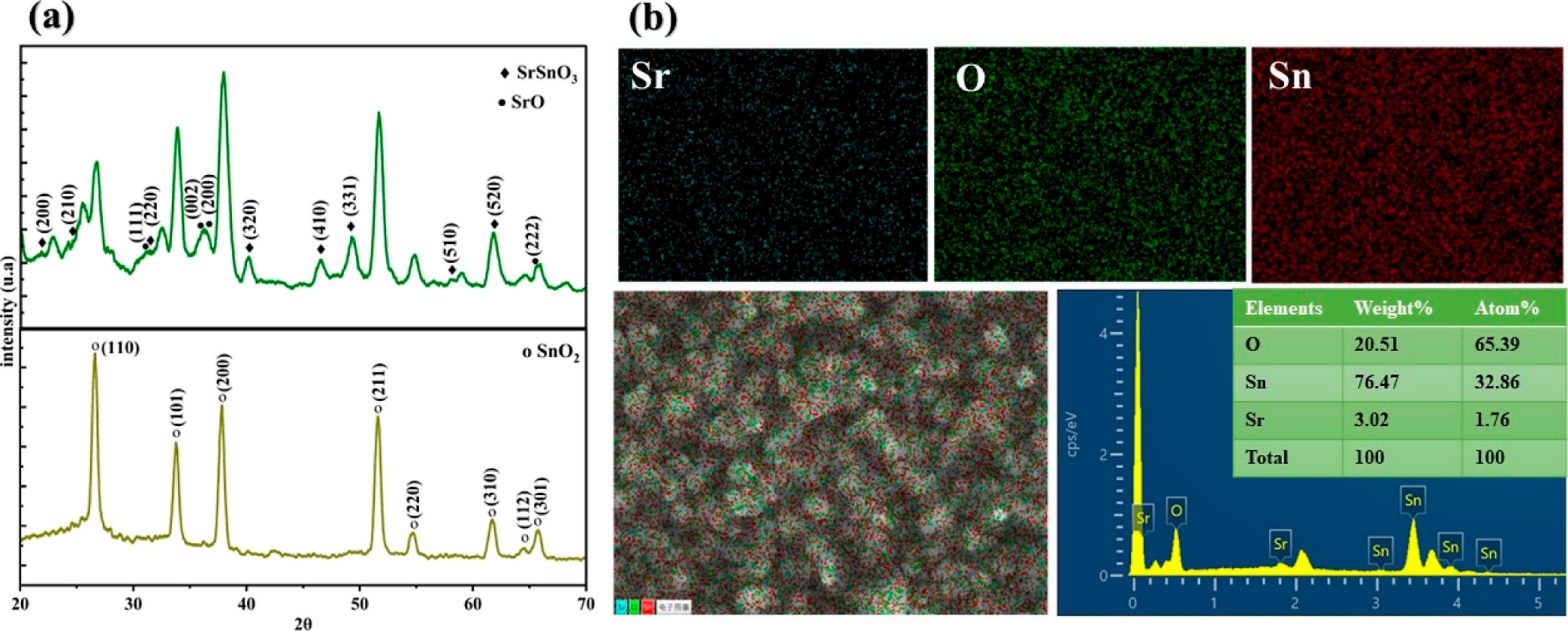
(a) XRD patterns of ETL before and after treatment. (b) Elemental mapping of the ETL.

In this study, we first used a chemical bath deposition (CBD) technique to uniformly deposit SnO_2_ onto the FTO substrate, providing complete uniform coverage. Then, the SSSO was synthesized using a spin-coating technique. This process involved depositing an aqueous solution containing Sr^2+^ ions on top of the previously deposited SnO_2_ layer using CBD. After deposition, all the layers on the FTO substrate were annealed at 180 °C for 1 h, which facilitated the formation of the required the SnO_2_–SSSO composite structure (Supporting Information, Section 3.1). Figure 1a presents the X-ray diffraction (XRD) profiles of the as-deposited SnO_2_ and SnO_2_–SSSO films, offering a comprehensive analysis of their crystal structures. This data was meticulously analyzed using MDI Jade software. For SnO_2_, specific peaks observed at 2θ values include 26.611, 33.89, 37.94, 51.78, 54.75, 61.87, 64.71, and 65.93. These values correspond to the crystal planes (110), (101), (200), (211), (220), (310), (112), and (301), respectively, as per the standard card PDF#41-1445.^45^ In contrast, SrSnO_3_ exhibits distinct peaks at 61.88, 58.27, 49.18, 46.35, 40.28, 31.32, 24.65, and 22.02, which correspond to the crystal planes (520), (510), (331), (410), (320), (220), (210), and (200), as referenced in PDF#22-1442. Furthermore, SrO displayed characteristic peaks at 31.55, 36.20, 36.57, and 65.39, corresponding to the crystal planes (222), (200), (002), and (111), as indicated in PDF#27-1304.^46,47^ The differences in peak intensity and location suggest variances in lattice parameters, concentrations, and crystallographic complexity when compared to SnO_2_.^48^Figure 1b illustrates the composition of the SnO_2_–SSSO layer using an energy-dispersive X-ray spectroscopy (EDX) pattern for the selected region diffraction. The EDX analysis of the SnO_2_–SSSO nanocomposite revealed signals attributed to the elements Sn, Sr, and O. Specifically, Sr emissions are identified by signals with energy levels of 0.2 and 1.8 keV, O emissions were characterized by a peak at 0.5 keV, and Sn emissions were distinguished by peaks at 3.1, 3.4, 3.9, and 4.4 keV. The compositional analysis demonstrated that the film consisted of oxygen (O), tin (Sn), and strontium (Sr) in proportions of 20.51, 76.47, and 3.02%, respectively, indicating a proportionate distribution.

**Figure 2:**
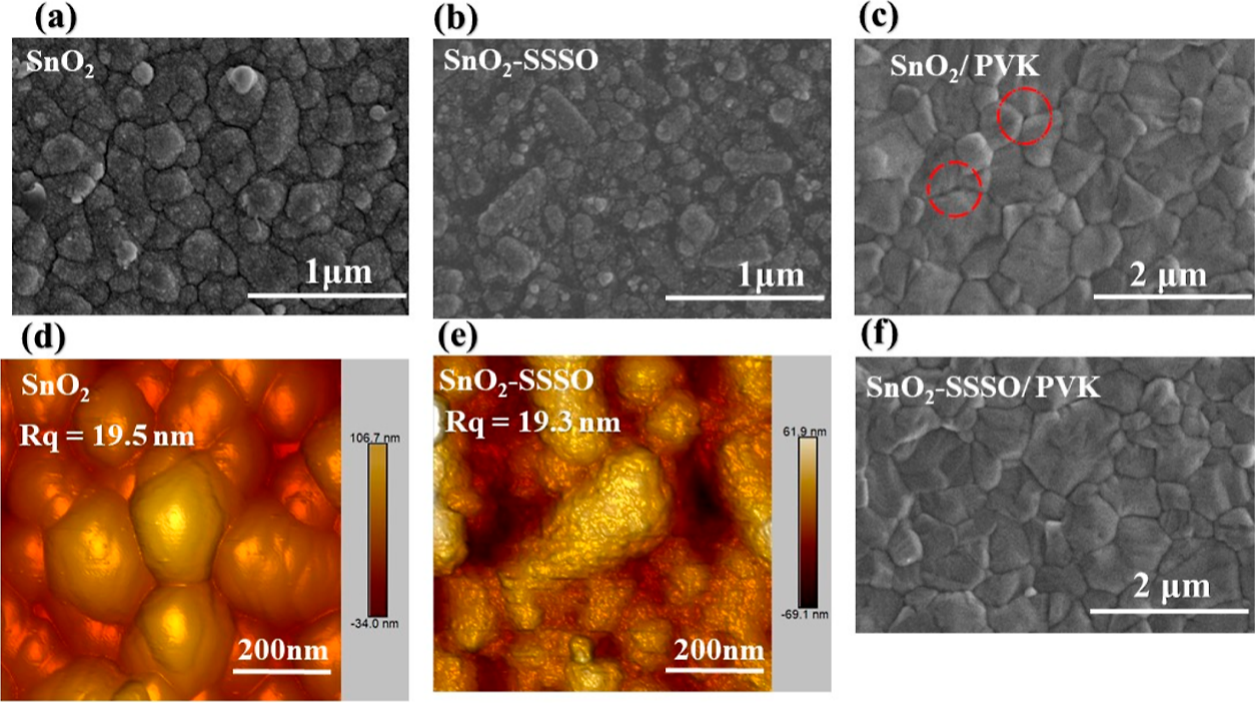
SEM images of (a) FTO/SnO_2_, (b) FTO/SnO_2_–SSSO, (c) FTO/SnO_2_/PVK, and (f) FTO/SnO_2_–SSSO/PVK films and AFM images of (d) FTO/SnO_2_ and (e) FTO/SnO_2_–SSSO.

**Figure 3:**
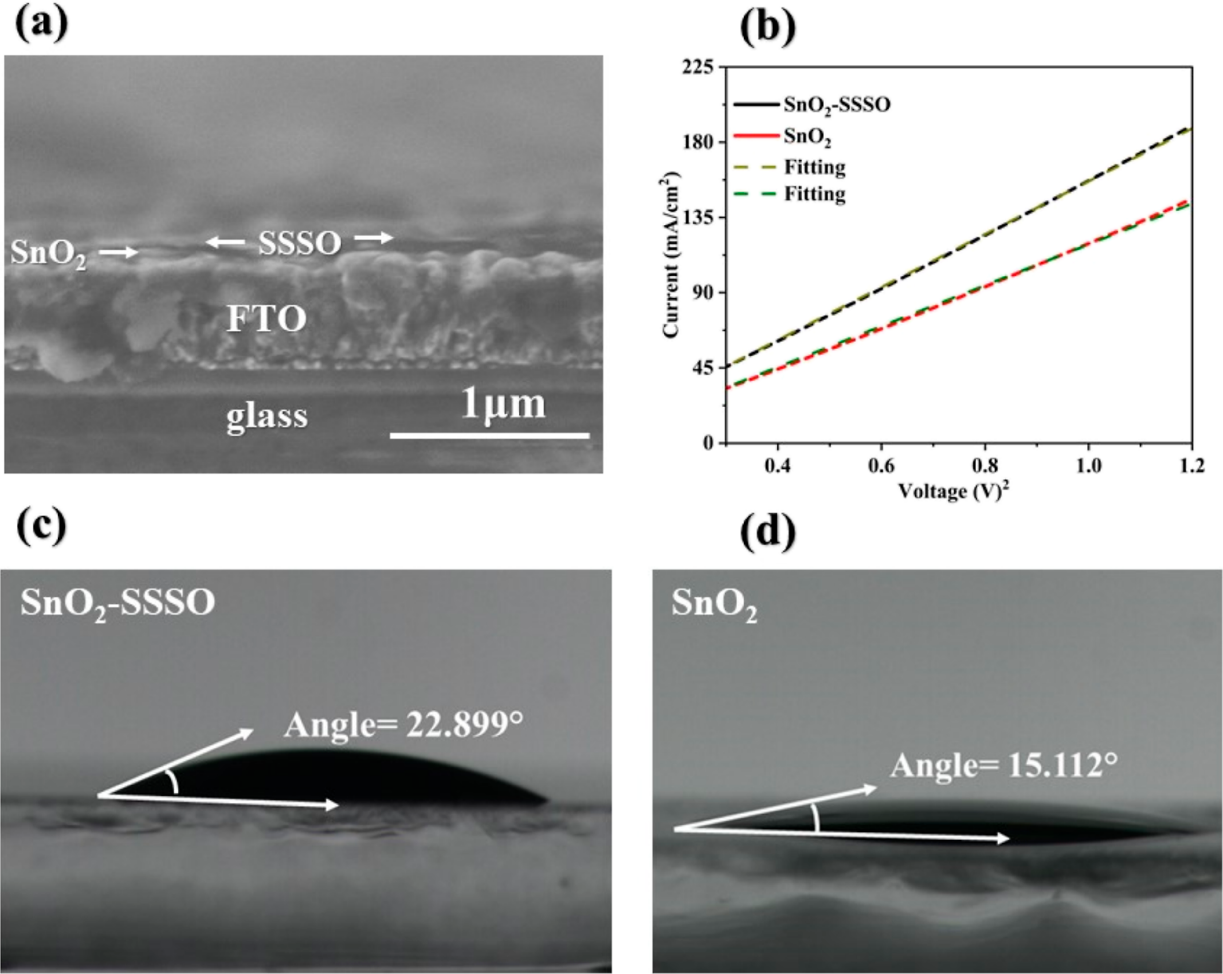
(a) SEM cross-section of FTO/SnO_2_–SSSO film, (b) *I*–*V* curves with the structure of FTO/ETL/Ag, and (c,d) angle contact for FTO/SnO_2_–SSSO and FTO/SnO_2_ films.

Scanning electron microscopy (SEM) and atomic force microscopy (AFM) were utilized to analyze the surface morphology of SnO_2_ films, both with and without the SSSO, in detail. SEM images, as presented in Figure 2a,b, revealed the differences in the morphologies of SnO_2_ and SnO_2_–SSSO films. The observed differences suggest that the SnO_2_–SSSO layer contributes to a thicker and denser surface compared with SnO_2_ films, which enhances electronic mobility and reduces recombination. Furthermore, AFM provided significant insights into the surface topography of SnO_2_ and SnO_2_–SSSO films, as depicted in Figure 2d,e. The SnO_2_–SSSO film exhibits a root-mean-square roughness (Rq) of 19.3 nm, slightly lower than the 19.5 nm measured for the SnO_2_ film. This reduction in roughness for the SnO_2_–SSSO film suggests a smoother substrate surface. Consequently, this smoother surface contributes to a more reliable connection between the ETL and the perovskite layer, leading to a decreased frequency of defects.^43,49^ The improvement in interface quality is crucial for the overall efficiency of PSCs. The hydrophobic properties, as shown in Figure 3c,d, along with reduced surface roughness, play a vital role in enhancing the interaction between the ETL and the perovskite, facilitating a more streamlined manufacturing process for perovskite layers. This enhanced contact is evidenced by the surface images of perovskite layers produced on SnO_2_ and SnO_2_–SSSO-coated substrates, as depicted in Figure 2c,f, respectively. The perovskite film fabricated on the SnO_2_–SSSO-coated substrate exhibits a larger average grain size compared to that on the SnO_2_-coated substrate. The statistical representation in Figure S1 shows a notable difference in the grain size, with perovskite grain diameters averaging 541.63 and 616.32 nm, respectively, in the absence and presence of SSSO modification. An increase in the grain size indicates a decrease in the number of grain boundaries, which reduces the likelihood of carrier recombination and minimizes nonradiative recombination losses at these boundaries.

To assess the electron mobilities of FTO/SnO_2_/Ag and FTO/SnO_2_–SrSnO_3_–SrO/Ag devices, we employed the space-limited charge current approach to demonstrate the capabilities of the ETL, as denoted by eq 1 and illustrated in Figure 3b. In this context, *L* represents the average thickness of the respective ETL films approximately 50 nm for the FTO/SnO_2_ film shown in Figure S4, and 35 nm for the FTO/SnO_2_–SSSO film shown in Figure 3a. ε_0_ is the permittivity of free space (ε_0_ = 8.85 × 10^–12^ c^2^/N m^2^) and ε_r_ is the dielectric constant, which was measured at 9^50^ for SnO_2_ and 15^51^ for SrSnO_3_.
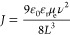
1

The electron mobility in the SnO_2_ film increases from 3.54 × 10^–6^ to 2.4 × 10^–5^ cm^2^ V^–1^ S^–1^ upon the incorporation of SSSO into ELT. This indicates that the addition of SSSO significantly enhances the electron mobility in the ETL. Consequently, the SnO_2_ films treated with SSSO exhibit significantly higher effectiveness for use in ETL compared to pure SnO_2_ films due to their enhanced electron mobility. As a result, in comparison to a pure SnO_2_ film, the SnO_2_ films treated with SSSO exhibit significantly higher acceptability for utilization in ETL due to their enhanced electron mobility. The improvement in mobility may be attributed to the presence of SrSnO_3_ within the ETL (SnO_2_), which possesses electronic properties that contribute to improving the movement of electrons, as explained by Tristan K.^51^ In addition, the SnO_2_ layer exhibits a surface contact angle of 15.112°. The contact angle increased to 22.899° due to a significant modification caused by the addition of SSSO to the SnO_2_ layer. Figure 3c,d illustrates a noticeable change in the contact angle, indicating an increase in the hydrophobic property. Enhancing hydrophobic property entails achieving a more consistent and efficient deposition of the perovskite layer, which is crucial for enhancing the quality and uniformity of perovskite crystals (Figure 2c,d).^52^

**Figure 4:**
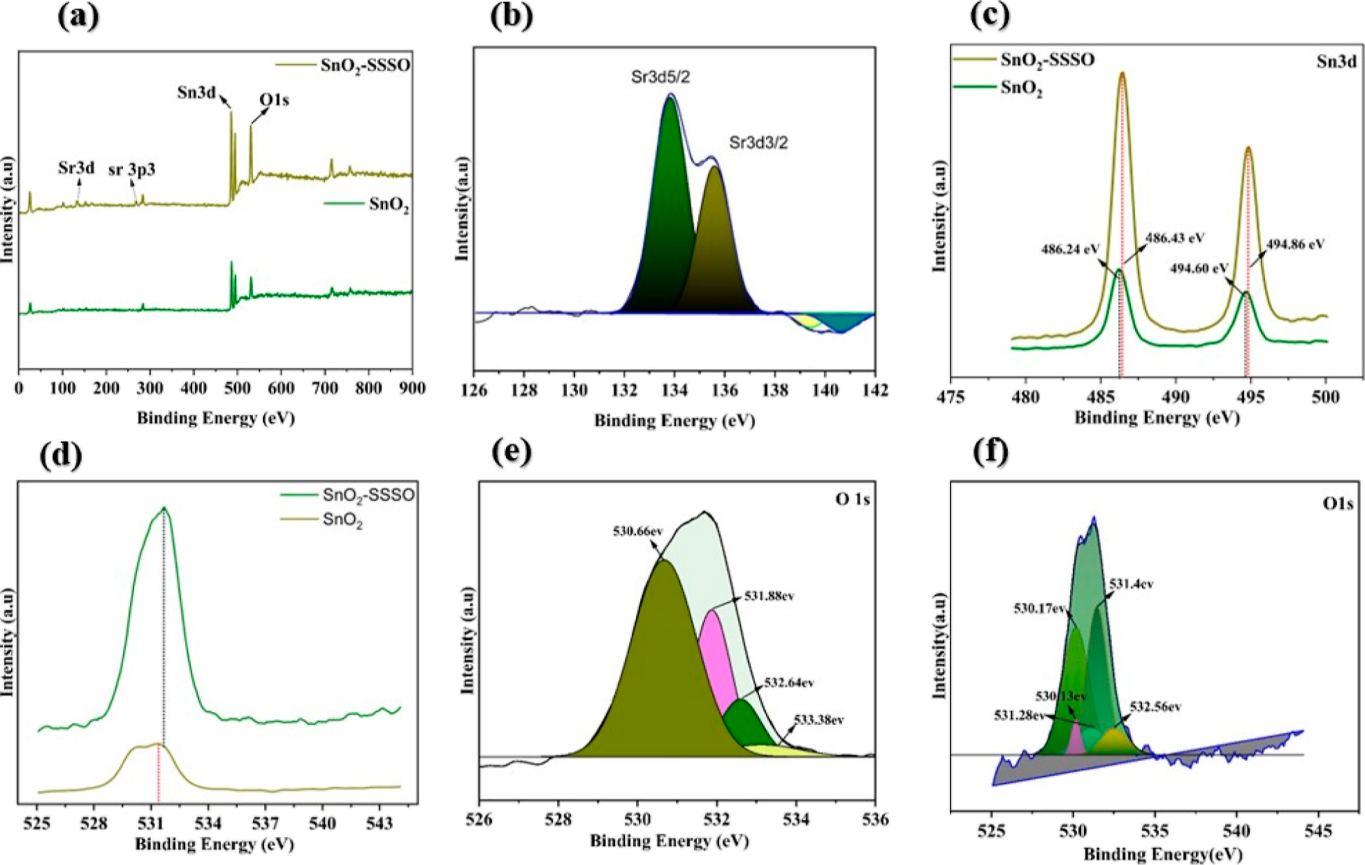
(a) XPS survey spectrum and the high-resolution XPS spectrum of (b) Sr 3d, (c) Sn 3d, and (d) O 1s for SnO_2_ and SnO_2_–SSSO and (e,f) O 1s.

X-ray photoelectron spectroscopy (XPS) was utilized to analyze the SnO_2_ and SnO_2_–SSSO films, aiming to confirm the presence of SrO. The C 1s peak at 284.6 eV served as a reference for adjusting the binding energy scales in the XPS data. First, the XPS survey spectrum, shown in Figure 4a, provided strong evidence for the composition and high purity of the synthesized SnO_2_ and SnO_2_–SSSO film nanocomposites. This analysis revealed distinct peaks corresponding to Sr, Sn, O, and C elements, with no noticeable impurities detected. Figure 4b illustrates that the XPS study identified the existence of the Sr^2+^ state in SrO within the complex SnO_2_–SSSO composition. The peaks corresponding to Sr 3d_5/2_ and Sr 3d_3/2_ were detected at approximately 133.8 and 135.6 eV, respectively. These peaks indicate the unique chemical state of SrO in this combination. In addition, XPS analysis revealed the presence of Sn^4+^ oxidation state inside the SnO_2_–SSSO complex.^53^ The binding energies for Sn 3d_5/2_ and Sn 3d_3/2_ typically range between 486.24 and 494.60 eV, respectively. After the deposition of SSSO, the positions of the Sn 3d_3/2_ and Sn 3d_5/2_ peaks in the SnO_2_–SSSO film shifted toward higher binding energies, specifically to 486.43 and 494.86 eV, respectively. The increase in binding energies suggests a specific interaction between SnO_2_ and Sr oxide molecules in the SnO_2_–SSSO film, as depicted in Figure 4c. Furthermore, Figure 4d illustrates the spectroscopic analysis of the SnO_2_–SSSO film, revealing a significant shift of the O 1s peak toward higher binding energy. This shift may indicate a reduction in the number of vacancies at the atomic sites of both Sn and O in the SnO_2_–SSSO film, compared to the standard SnO_2_ film. The decrease in vacancies offers compelling evidence for the integration of Sr oxide into the structural framework of SnO_2_. The XPS spectra for the O 1s, as shown in Figure 4e, display prominent and asymmetrical bands. After deconvolution of the peak for the SnO_2_–SSSO nanocomposite, distinct O 1s peaks emerge at energy levels of 530.66, 531.88, 532.64, and 533.3 eV. Those peaks represent lattice oxygen (OV), oxygen vacancy regions (OL), and chemisorbed oxygen/water species, respectively. In addition, the XPS spectra for O 1s depicted in Figure 4f exhibit broad and irregular bands upon deconvolution of the SnO_2_ O 1s peak. This analysis identifies characteristic O1 peaks at energy levels of 530.17, 530.14, 531.28, 531.4, and 532.56 eV. Finally, the XPS analysis of the SnO_2_–SSSO composite unequivocally reveals the presence of specific elements and their oxidation states, which is consistent with those found in SnO_2_, SrSnO_3_, and SrO. This conclusion is supported by the characteristic peaks of strontium (Sr), tin (Sn), and oxygen (O), alongside the identification of Sr in the Sr^2–^ state and Sn in the Sn^4+^ state, which is characteristic of SrSnO_3_, SrO, and SnO_2_, respectively. When combined with XRD analysis, these observations firmly confirm that the SnO_2_–SSSO complex is indeed composed of these three oxides, each present in its expected form.

**Figure 5:**
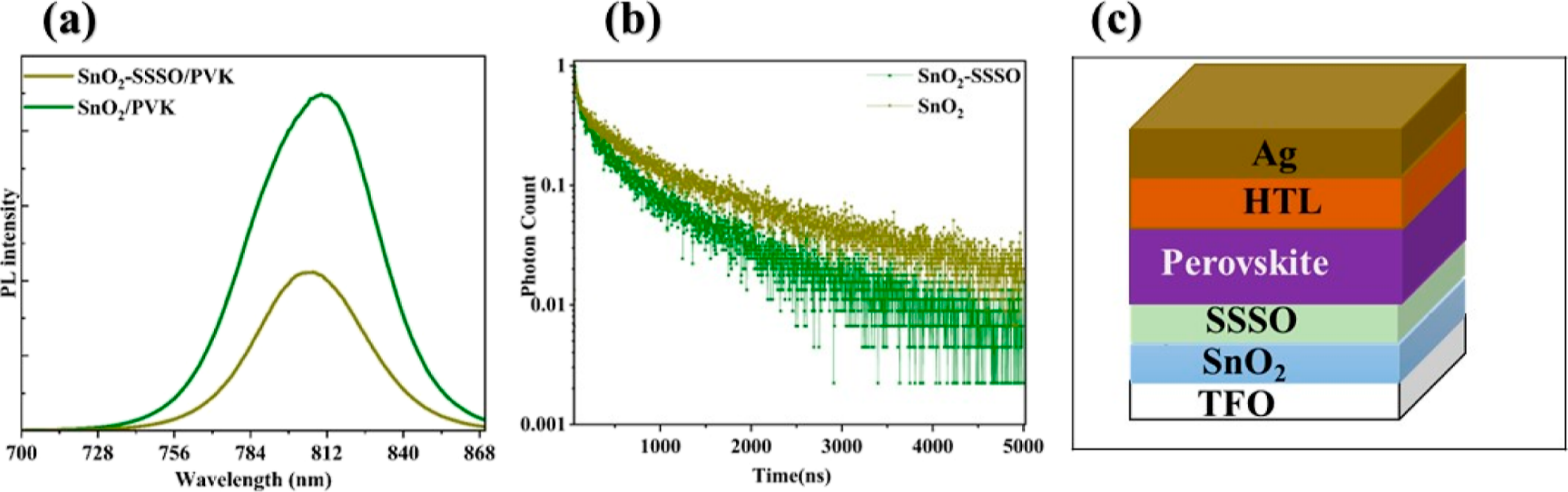
(a) PL and (b) TRPL spectra of PVK film embedded with SnO_2_/PVK and SnO_2_–SSSO/PVK. (c) PSC’s structure.

In order to investigate the effects of protecting the absorption layer in the perovskite film with SSSO and evaluate the improvement in charge separation and recombination at the ETL/perovskite interfaces, we employed steady-state photoluminescence (PL) analysis. This method was used to study the kinetics of electron transfer. The PL of the perovskite film in a stable condition was measured, as illustrated in Figure 5a. The PL peaks for both samples appeared at similar positions, however, the PL intensity of the perovskite film placed on the SnO_2_–SSSO film was lower than that observed on the SnO_2_ film. The result suggests that the SnO_2_–SSSO layer is less effective in hindering the formation of photogenerated carriers. In perovskite materials, a decrease in PL intensity typically indicates enhanced charge carrier separation. This implies that carriers are rapidly transported away from the light-absorbing layer, thereby reducing the time available for recombination and light emission. This effect may be attributed to the high dielectric stability of SrO, which is effective in passivating the surface of perovskite materials. Consequently, this reduces the density of surface states that can serve as recombination centers, as mentioned by Tripkovic.^54^

Figure 5b displays the systematic development of PL spectra for the perovskite material deposited on both the SnO_2_ film and SnO_2_–SSSO film. To quantify the photon decay over time, we employed eq 2, which characterizes the observed decay in PL.
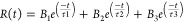
2

This equation involves three distinct time constants measured in nanoseconds: τ_1_ for a short duration, τ_2_ for an intermediate duration, and τ_3_ for a longer duration. As detailed in Table S1, for both the processed films (SnO_2_–SSSO/PVK) and the reference films (SnO_2_/PVK), the PL lifespan (τ) of the SnO_2_/PVK film was measured at 510.30 ns. In contrast, the treated film (SnO_2_–SSSO/PVK) exhibited a significantly reduced lifetime of 272.56 ns. This reduction in the PL lifetime of the SnO_2_–SSSO/PVK film suggests enhanced charge extraction properties compared to the SnO_2_/PVK film. Consequently, it implies that the SnO_2_–SSSO/PVK film has a greater ability to convert absorbed light into electrical energy.

**Figure 6:**
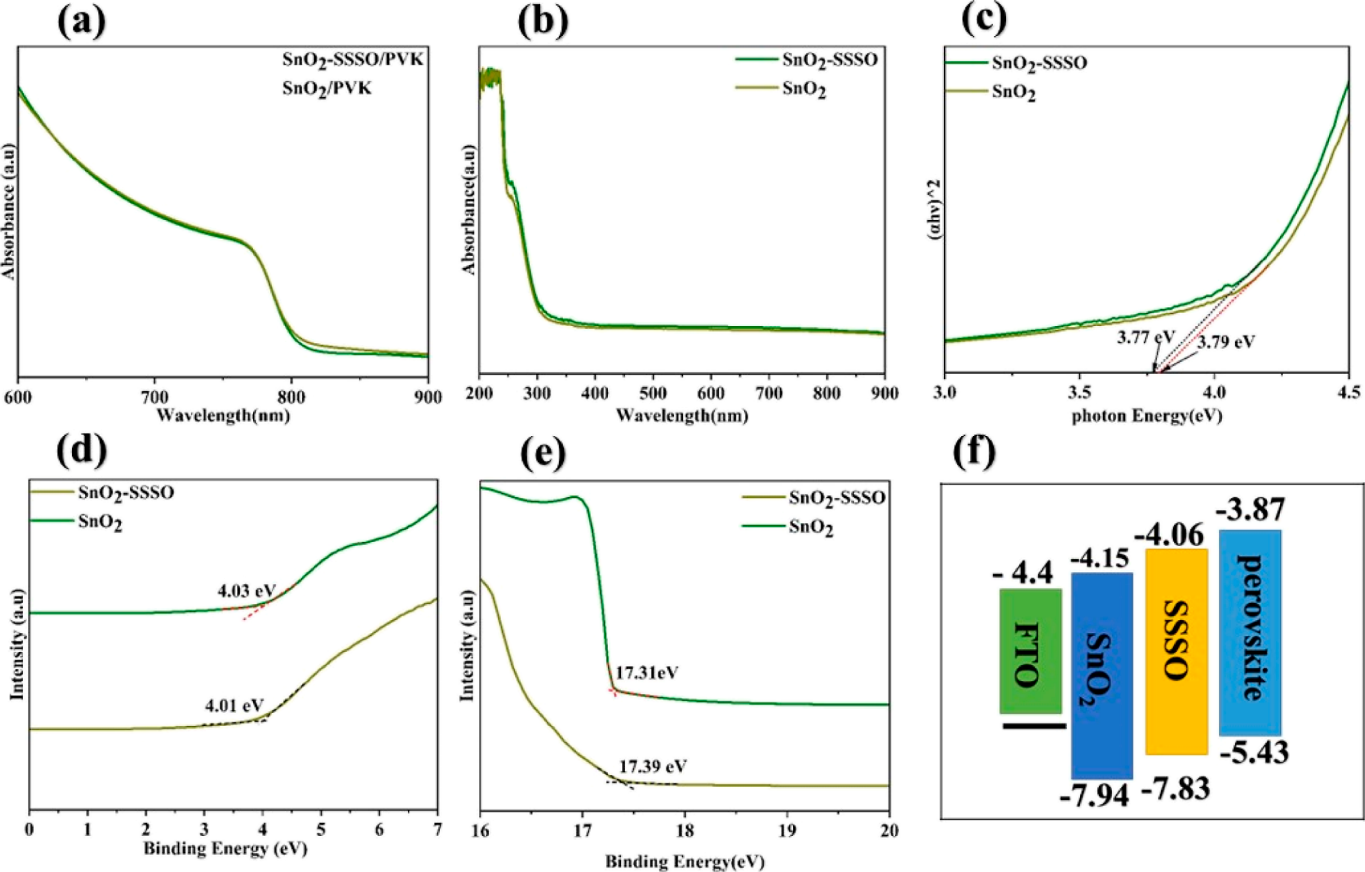
UV–vis spectra of (a) FTO/SnO_2_/perovskite and FTO/SnO_2_–SSSO/perovskite films and (b) pristine FTO/SnO_2_ and FTO/SnO_2_–SSSO films, (c) Tauc plots for pristine FTO/SnO_2_ and treated FTO/SnO_2_–SSSO films, and (d) and (e) UPS spectra for pristine FTO/SnO_2_ and treated FTO/SnO_2_–SSSO films in low- and high-energy regions. (f) Diagram of energy band alignment for perovskite devices.

To thoroughly understand the impact of the SSSO on the electrical and optical properties of the films, we performed UV photoelectron spectroscopy (UPS) and UV–visible spectral analyses on SnO_2_ films both with and without the SSSO. Figure 6b displays the UV–vis spectra of FTO/SnO_2_ and FTO/SnO_2_–SSSO films, respectively. It shows that the FTO/SnO_2_–SSSO film is only slightly more absorbed than the FTO/SnO_2_ film in the UV region because the layer containing Sr is too thin. However, when the thickness of the processed film was increased to 120 nm (Figure S5b), we observed significantly higher absorption (Figure S5a). This enhanced performance can be attributed to SrSnO_3_’s capacity to absorb UV radiation, this capability originates from its distinct electronic and structural properties.^55^

In addition, the UPS tests, as depicted in Figure 6d,e provide insights into the Fermi edge (*E*_D_) and cutoff binding energy (*E*_cutoff_), essential for assessing the work function (WF) and valence band characteristics of the FTO/SnO_2_ and FTO/SnO_2_–SSSO films, respectively. To calculate the WF (or Fermi levels), the position of the conduction band minimum (ECBM), and the valence band maximum (EVBM) of both FTO/SnO_2_–SSSO and pure FTO/SnO_2_ films, we utilized formulas 3–5.
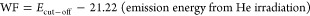
3
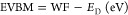
4
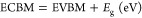
5

The Fermi edge and binding energy, depicted in Figure 6d,e, indicate that the maximum energy of the valence band (EVBM) for FTO/SnO_2_ and FTO/SnO_2_–SSSO, calculated using formula 4, is found to be −7.94 and −7.83 eV, respectively. Moreover, applying eq 5 reveals that the lowest energy level of the conduction band (ECBM) is determined to be −4.15 eV for FTO/SnO_2_ and −4.06 eV for FTO/SnO_2_–SSSO films, as detailed in Table S2 of the Supporting Information. The band gap energies (*E*_g_) of FTO/SnO_2_ and FTO/SnO_2_–SSSO were calculated using the Tauc plot technique. The values obtained were 3.79 and 3.77 eV, respectively, as shown in Figure 6c. In Figure 6f, we displayed an energy band alignment diagram for the devices being studied. The EVBM, ECBM, and band gap data for PVK were obtained from our previous works^33^ and FTO.^56^ The energy band alignment diagram presented in this study illustrates a significant improvement in the performance of PSCs through the incorporation of an SSSO as both a passivating layer at the interface and a UV filter. The conduction band energy level (ECB) in the FTO/SnO_2_–SSSO film aligns more compatibly with the energy level of the PVK layer compared to the ECB in the pure FTO/SnO_2_ film. This alignment enhances the electron injection force from the PVK layer to the ETL. Moreover, this compatibility facilitates the efficient extraction of photogenerated carriers from the PVK layer, ensuring their seamless transport to the ETL.

**Figure 7:**
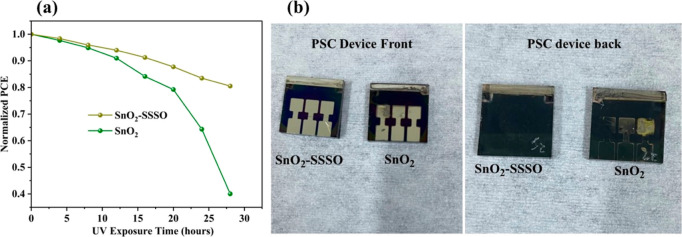
(a) Stability assessment of perovskite devices under UV radiation (285 nm, 1.63 mW/cm^2^) at approximately 25 °C in an air atmosphere (RH 45–50%) and (b) UV impact on treated and untreated devices.

In order to verify whether the new layer can protect the absorbent layer from the effects of UV rays, UV exposure experiments were conducted on both treated and untreated devices. Continuous UV radiation was simulated using a UV lamp, with both the control and treatment devices exposed concurrently. Their performance was evaluated at 4 h intervals over a 30 h period, as depicted in Figure 7a. The results showed that the treated devices maintained an effectiveness of 17.64% after 30 h under UV rays, compared to the initial 21.90% before exposure. In contrast, untreated devices initially recorded an efficacy of 17.92%, but experienced a significant drop in performance after UV exposure, falling to 7.17%. This decline was notably accompanied by degradation in the perovskite layer, observable on both the rear and front sides of the device, as illustrated in Figure 6b. Conversely, the treated devices showed no such degradation, highlighting their enhanced resistance to UV-induced damage. This superior performance is attributed to SrSnO_3_ ability to absorb UV rays and modulate light emission, a capability stemming from its unique electronic and structural properties, as emphasized in studies by W.F. Zhang et al.^55^ and Weifeng Zhang et al.^57^

**Figure 8:**
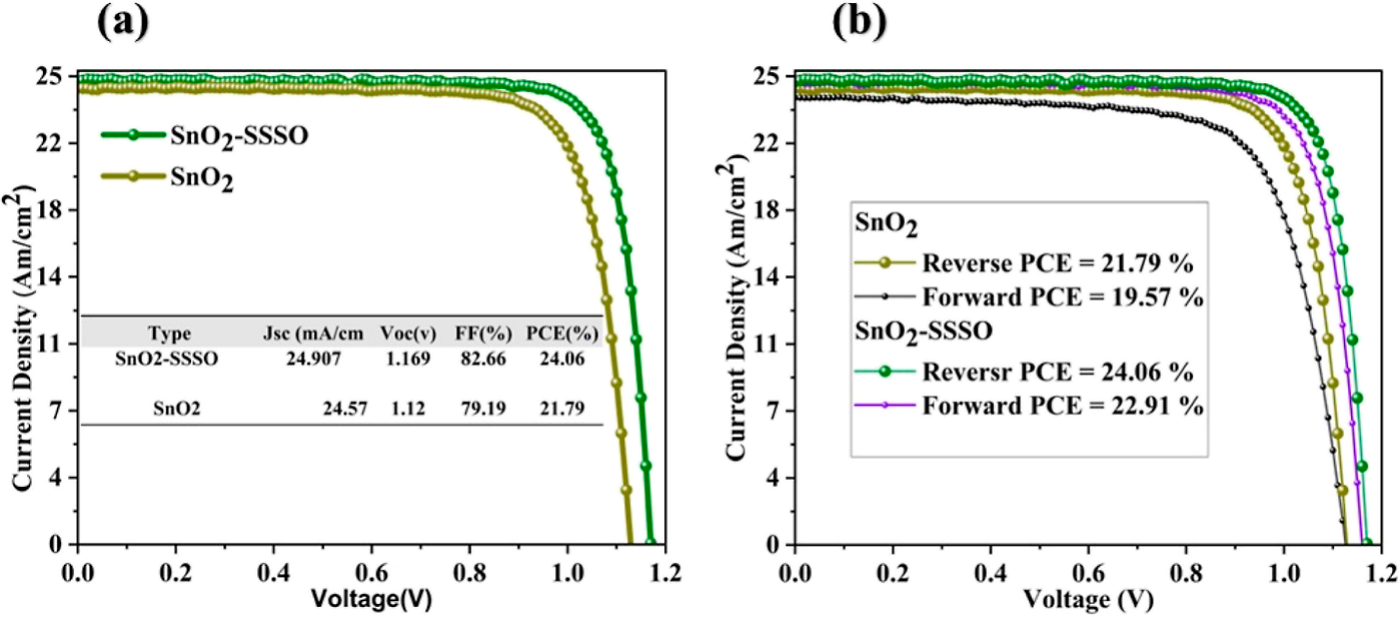
(a) *J*–*V* curves for pristine devices FTO/SnO_2_ and treatment devices FTO/SnO_2_–SSSO and (b) hysteresis analysis at a 50 mV/s scan rate and 0.0652 cm^2^ mask area.

In this research, our primary focus was on enhancing the capabilities and improving the performance of perovskite solar cell devices, primarily by interface passivation and UV ray filtration. This was achieved by incorporating SSSO on top of the ETL (SnO_2_). We conducted a series of experiments to fabricate PSCs with a structured configuration designated as FTO/ETL/PVK/HTL/Ag. These PSCs demonstrated a PCE of 24.06%, a current density (*J*_sc_) of 24.90 mA/cm^2^, an open-circuit voltage (*V*_oc_) of 1.16 V, and a FF of 82.66%, as shown in Figure 8a. In comparison, the reference devices exhibited a PCE of 21.79%, a *J*_sc_ of 24.57 mA/cm^2^, an FF of 79.19%, and a *V*_oc_ of 1.12 V, as indicated in Figure 8a and Table 1. Additionally, it is important to note that the hysteresis index, calculated as (PCEreverse – PCEforward)/PCEreverse, has decreased from 10.92 to 4.77%, as detailed in Table S3 and Figure 8b. This reduction suggests that charge stacking and carrier recombination, typically caused by interface defects, have been mitigated. This improvement is attributed to the presence of SrO, which effectively suppresses interfacial defects between the PVK layer and the ETL, leading to enhanced charge extraction and hysteresis suppression.^43,58^

**Table 1:** Photovoltaic Parameters of Untreated FTO/SnO_2_ PSCs and Strengthened FTO/SnO_2_–SSSO PSCs

type	PCE (%)	*V*_oc_ (%)	FF (%)	*J*_sc_ (mA/cm^2^)
SnO_2_	21.79	1.128	79.19	24.57
SnO_2_–SSSO	24.06	1.16	82.66	24.90

**Figure 9:**
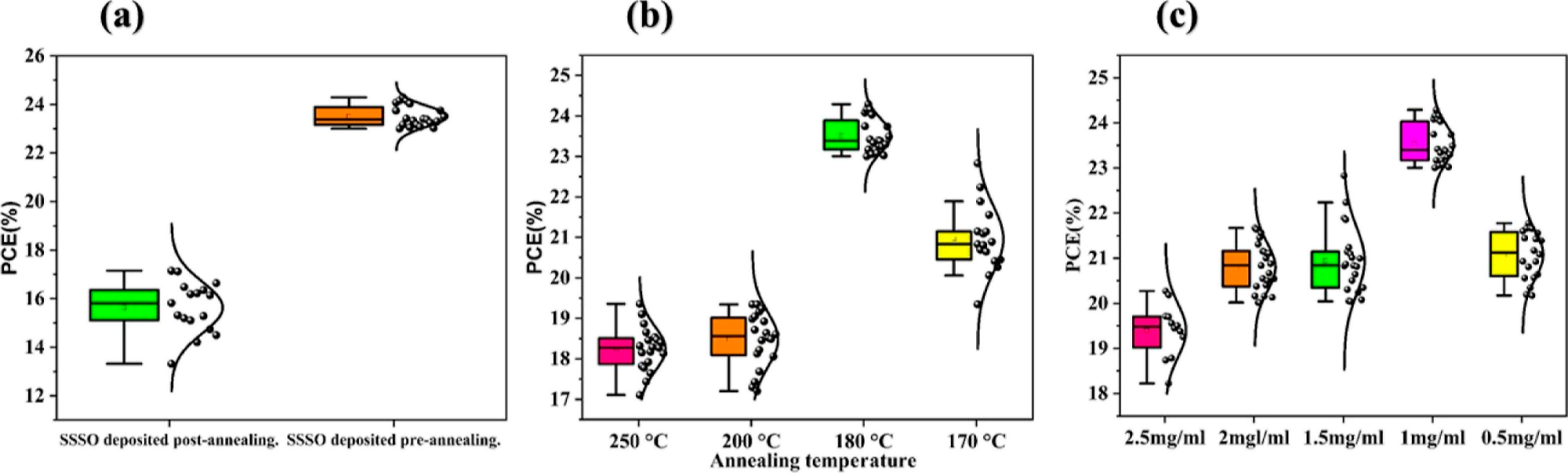
(a) Comparison of PCE for treated films (SnO_2_–SSSO) depositing before and after the annealed SnO_2_ layer, (b) variations in the PCE for treated films (SnO_2_–SSSO) annealed at different temperatures, and (c) peak PCE values of treated films (SnO_2_–SSSO) at various concentrations.

The deposition of SSSO onto the top of the ETL (SnO_2_) was meticulously planned to encompass a range of concentrations, accompanied by annealing processes at various temperatures. This systematic technique facilitated a comprehensive investigation into how these factors influence the overall performance of the devices, with particular emphasis on the effects observed before and after the annealing process. Initially, the performance of the devices was assessed by examining the deposition of SSSO onto the top of the ETL (SnO_2_) both before and after annealing. More specifically, after depositing the ETL (SnO_2_) via the chemical bath method, it was directly annealed at 170 °C, as detailed in a previous study (see the Supporting Information).^1^ Following this, SSSO was deposited onto the ETL (SnO_2_), and the resulting device, FTO/SnO_2_–SSSO, was then annealed again at a different temperature. However, the performance of the device was found to be extremely weak, as illustrated in Figure 9a (SSSO deposition post-annealing). The second step involved depositing the SSSO onto the FTO/SnO_2_ substrate, which had been prepared by the chemical bath method, before annealing it. The obtained device (FTO/SnO_2_–SSSO) was then annealed at various temperatures (250, 200, 180, and 170 °C), as shown in Figure 9b. It was discovered that depositing the SSSO onto the ETL (SnO_2_) before annealing was effective, with the devices exhibiting optimal performance at 180 °C, as indicated in Figure 9a (SSSO deposited preannealing). As a result, the PCE was calculated at different concentrations of SSSO (2.5, 2, 1.5, 1, and 0.5 mg/mL), as shown in Figure 9c. The optimal concentration of SSSO was determined to be 1 mg/mL, at which the PCE reached its highest level.

**Figure 10:**
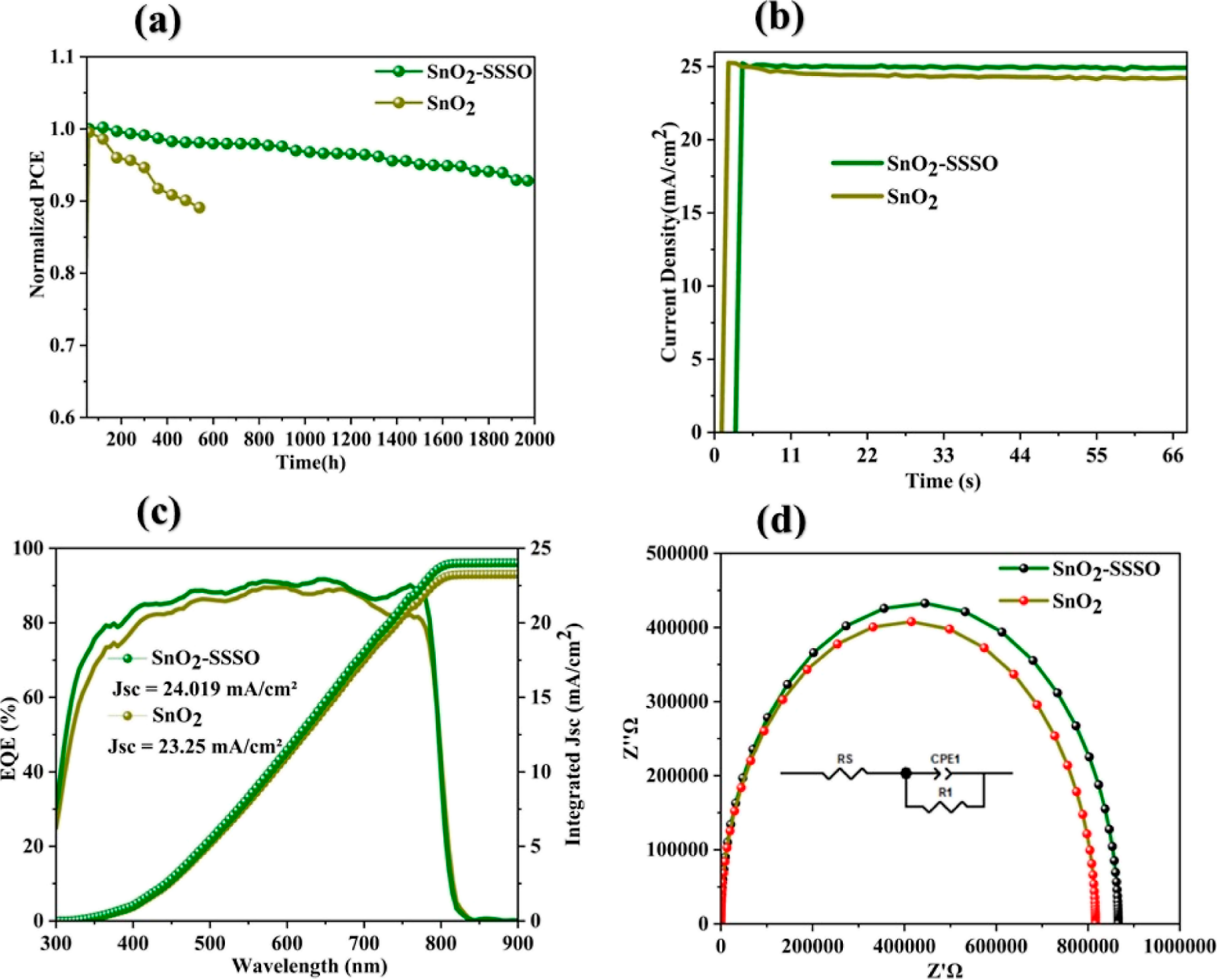
(a) Stability assessment of perovskite devices, (b) steady-state maximum power point determination for pristine and treated films, (c) IPCE spectra (left) and the corresponding integrated *J*_sc_ (right) are derived based on the IPCE data, and (d) Nyquist plots of PSCs with or without SSSO.

PSCs devices underwent a comprehensive 2000 h stability study to evaluate the effect of UV filtering and passivation on their performance. The devices were tested at 60 h intervals and stored under controlled conditions at room temperature, maintaining a humidity level between 5 and 8%. The initial PCE of the treated perovskite devices decreased from 24.15 to 22.50% in less than 2000 h, as shown in Figure 10a, while the reference device exhibited a decrease from 21.79 to 17.83% within 580 h, also illustrated in Figure 10a. These results underscore the significant roles of SrO and SrSnO_3_ in diminishing facial blemishes and filtering UV rays, respectively. Figure 10b illustrates the peak level of sustained power point tracking performance achieved by the devices when processed under AM1.5G illumination. The treated devices exhibited little change from their initial state, whereas the untreated devices demonstrated a noticeable decline in comparison over the same period. As illustrated in Figure 10c, the external quantum efficiency of electrons increases due to enhanced electrical conductivity and electron mobility, resulting in improved electron extraction efficiency. As a result, the short-circuit current density (*J*_sc_) of devices incorporating SSSO is higher than that of devices lacking SSSO, which is consistent with the findings from the *J*–*V* measurement analysis. The Nyquist curve, as depicted in Figure 10d, showcases the impedance responses of perovskite devices with and without SSSO, corresponding with the equivalent circuit diagram in the figure’s inset. The relevant parameters are detailed in Table S4. It was observed that PSCs treated with SSSO exhibited lower series resistance (*R*_s_, 138.4 Ω) compared to untreated cells (227.2 Ω), suggesting improved conductivity and decreased resistive losses. This improvement can be attributed to the SSSO layer facilitating a more efficient charge transport pathway, thereby minimizing resistive losses that can adversely affect the device’s overall performance.^59−61^ Furthermore, SSSO-treated cells showed a higher recombination resistance (*R*_rec_, 865,780 Ω) than untreated cells (815,890 Ω), indicating reduced charge carrier recombination and, consequently, enhanced efficiency.^59−61^ Nonradiative recombination is a critical loss mechanism in PSCs, where charge carriers recombine without contributing to the electrical output. By limiting this process, SSSO treatment enhances the charge carrier lifetime, leading to improved device efficiency.^59−61^

**Figure 11:**
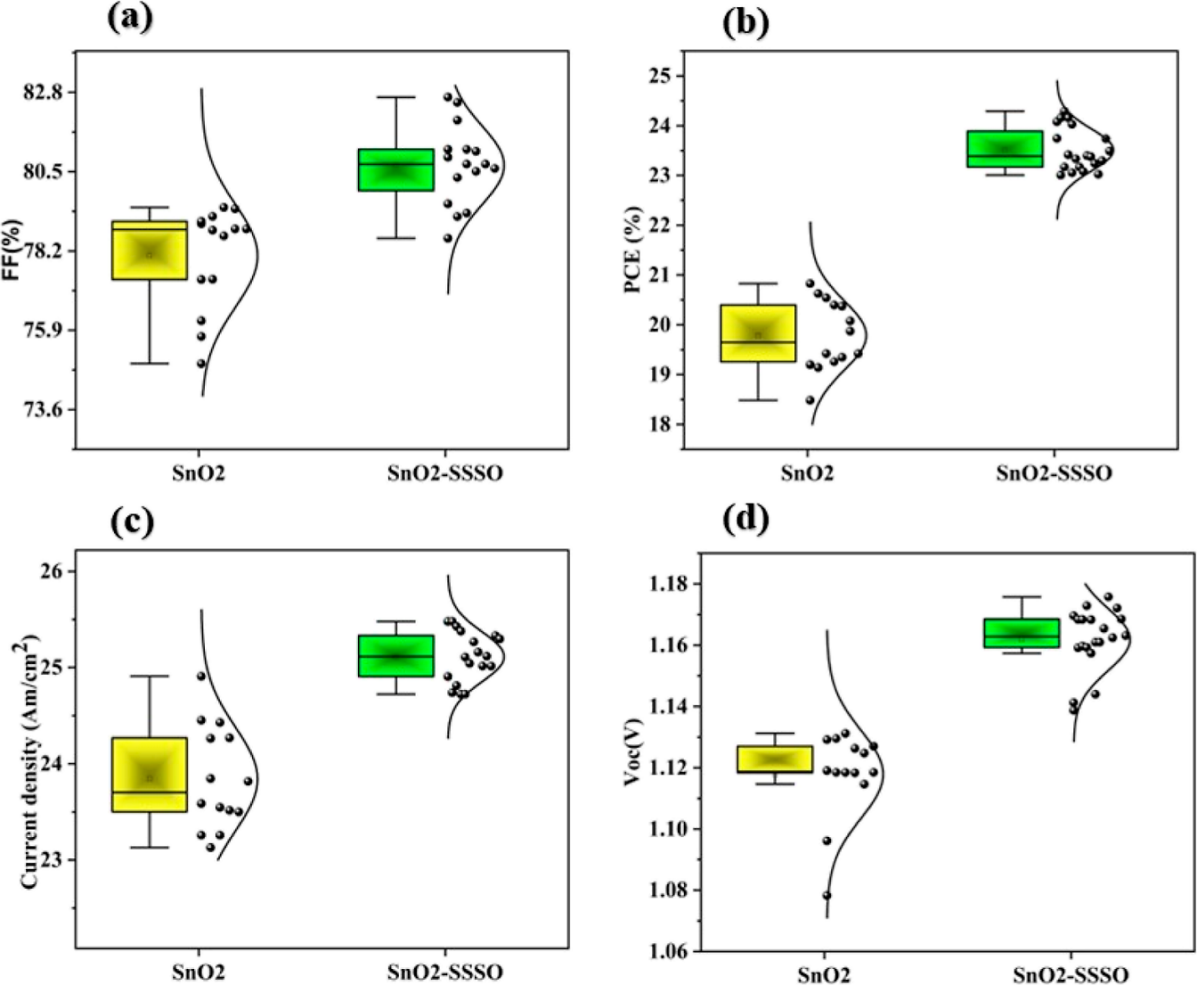
(a–d) Comparison of FF, PCE, *J*_sc_, and *V*_oc_ between pristine SnO_2_ and SnO_2_–SSSO treated films.

Figure 11a–d presents an insightful visual representation of distribution Box plots effectively analyze performance variations across a data set of 15 distinct devices, for both SnO_2_ and SnO_2_–SSSO films. These illustrations offer a comprehensive examination of key parameters, including FF in the Figure 11a, PCE in the Figure 11b, *J*_sc_ in the Figure 11c, and *V*_oc_ in the Figure 11d. A particularly notable aspect of this analysis is the introduction of SSSO, which serves a dual purpose: it passivates the interface and filters UV rays. Its inclusion is correlated with higher PCE values and, more remarkably, with substantial improvements in performance reproducibility compared to devices lacking the SSSO component. These findings have significant implications for the practical applications of SnO_2_–SSSO ETL in perovskite solar cells.

## Conclusions

In conclusion, this study underscores the enhancement of perovskite solar cell performance through UV filtration and defect passivation at the SnO_2_/PVK interfaces. This innovative approach boosts PSCs performance by combining SnO_2_-enhanced electron transfer, SrO-mediated interface defect passivation, and SrSnO_3_’s UV radiation filtering capability. The efficacy of this method is highlighted by significant improvements in several parameters. An increase in *V*_oc_ from 1.12 to 1.16 V was observed, alongside an enhancement in FF from 79.4 to 82.66%, a rise in *J*_sc_ from 24.5 to 24.9 mA/cm^2^, and most notably, a significant boost in the PCE of PSCs from 21.79 to 24.06%. Furthermore, SnO_2_–SSSO-treated perovskite solar cells demonstrated remarkable stability, with only a minor reduction in the PCE from 24.15 to 22.50% over approximately 2000 h. This is in stark contrast to the untreated SnO_2_ PSCs, which exhibited a substantial decrease in efficiency from 21.79 to 17.83% within just 580 h. Anyway, the treatment of PSCs with SSSO emerges as a crucial enhancement strategy, offering the dual benefits of significantly improved efficiency and stability.

## Supporting Information Available

Materials, fabrication of SnO_2_ and SnO_2_–SSSO films, device fabrication, characterization and measurements, particle size of SnO_2_–SSSO, TRPL fitting curve for the SnO_2_ and SnO_2_–SSSO films, cross-sectional SEM of FTO/SnO_2_ film, UV–vis of FTO/SnO_2_ and FTO/SnO_2_–SSSO before and after deposition PVK layer, cross-sectional SEM of FTO/SnO_2_, TRPL property values for pristine SnO_2_ PSCs and treated SnO_2_–SSSO PSCs films, table of the photovoltaic characteristics of pristine SnO_2_ PSCs and SnO_2_–SSSO PSCs, table of photovoltaic parameters of devices in both reverse and forward scan directions using SnO_2_ and SnO_2_–SSSO films, and table of the fitting parameters obtained from the EIS data of devices based on SnO_2_ and SnO_2_–SSSO film (PDF)
