# Supplementary material for: Improving the Efficiency and Stability of Perovskite Solar Cells by Refining the Perovskite-Electron Transport Layer Interface and Shielding the Absorber from UV Effects
Source: ACS Appl Mater Interfaces. 2024 May 27;16(22):28493–504. doi: 10.1021/acsami.4c03329 (PMC11163405; doi:10.1021/acsami.4c03329)
Supplement: Supplementary file 1 — am4c03329_si_001.pdf [file am4c03329_si_001.pdf]

Supporting Information

Improving the Efficiency and Stability of Perovskite Solar Cells by Refining the Perovskite-Electron Transport Layer Interface and Shielding the Absorber from UV Effects

Salah AL-Shujaa,[1] *Peng Zhao,*[1] *Dingqian He,*[1] *Basheer Al-Anesi,*[4] *Yaqing Feng,*[1] *Jianxing Xia,*[2*] *Bao Zhang,*[1, 3*] *Yi Zhang.*[1, 2, 3*]

[1] School of Chemical Engineering and Technology, Tianjin University, Tianjin 300350, China.

[2] Institute of Molecular Plus, Tianjin University, Tianjin 300072, China.

[3] Haihe Laboratory of Sustainable Chemical Transformations, 300192 Tianjin, China.

[4] Faculty of Engineering and Natural Sciences, Tampere University, Tampere, 33014, Finland.

E-mail addresses: xiaban@tju.edu.cn (Jianxing Xia); baozhang@tju.edu.cn (Bao Zhang); yi.zhang@epfl.ch (Yi Zhang)

1. Materials

Fluorine-doped tin oxide (FTO) coated glass, exhibiting a sheet resistance of 8 Ω/sq, was procured from Pilkington. DI water and hydrochloric acid (HCl) at a concentration of 37% weight percent in water and urea were sourced from Tianjin Jiangtian Chemical Technology Co. Tin (II) chloride dihydrate ($SnCl_2 \cdot 2H_2O$) with a purity of 99.995% was sourced from 3Amaterials and used without further processing. Also, $Sr(NO_3)_2$ with purity of 99.995% was bought from Sigma and used as received.

2. Chemical Bath Deposition (CBD) of the SnO_2 layer

Based on Jason J. Yoo's earlier research[1], with certain modifications, the experiment was conducted as follows:

A. Cleaning the FTO Devices. The FTO substrate was subjected to a comprehensive cleaning process. This involved sequential sonication for 20 minutes in various solutions: Hellmanex (to remove organic contaminants), deionized water (to rinse off residual chemicals), acetone (for degreasing), and isopropanol (IPA) for final cleaning.

B. Preparation of the CB Solution. CB solution was prepared. A 250 ml glass beaker had been filled with 75 ml of distilled water. Next, 935 mg of urea was added, and the mixture was agitated at 25 °C until complete dissolution. Then 207 mg of tin(II) chloride dehydrate ($SnCl_2 \cdot 2H_2O$) was introduced. Stirring continued until the solution became transparent. Following this, 935μL of hydrochloric acid (HCl) and 18.5μL of thioglycolic acid (TGA) were added, one after the other. Each addition was accompanied by 10 minutes of agitation, ensuring that the mixture remained homogeneous.

C. Preparation of FTO devices. A small adhesive strip was accurately attached to the area containing the battery's anode. This specific spot is where the fluorine-doped Tin Oxide (FTO) substrate made direct contact with silver Ag or Au. The FTO was then meticulously positioned in a container suitable for Hellendahl staining or any other kinds, as depicted in Figure 1(c) of the manuscript.

D. In the final stage. In the final stage, the FTO substrates, prepped as per step C, were placed in a Hellendahl staining dish and then into a glass beaker holding the CB solution from step B. This assembly was then submerged in an oil bath heater, with the reaction kickstarting at a steady 95°C. Once the target pH was reached (1.5), the process was halted, and the substrates were extracted. Following this, the FTO/SnO_2 glass substrates underwent a thorough cleaning, being sonicated in

DI water and ethanol for five minutes each. After washing, the subsequent step was a 5-minute thermal drying process in an oven. After the drying phase, the substrate underwent a 15-minute exposure to ozone (O_3). Preparation for the deposition of the new layer (SnO_2-SSSO).

3. Fabrication FTO/SnO_2/SSSO films.

First, the experimental procedure began with the accurate preparation of $Sr(NO_3)_2$ aqueous solutions at various concentrations: 2.5, 2, 1.5, 1, and 0.5 mg/mL. These solutions were continuously stirred for 3 hours at room temperature, followed by thorough filtration using a 0.22µm syringe filter to ensure purity and preparedness for the next step.

Second, after subjecting FTO/SnO_2 to a 5-minute thermal drying process in an oven and then being exposed to ozone (O_3) for 15 minutes. The filtered solutions were then uniformly deposited onto the FTO/SnO_2 substrate using a spin-coating technique, which involved rotating at 4000 rpm for 30 seconds.

In the final phase of the process, the coated substrates were carefully annealed at 180°C for 1 hour under atmospheric conditions without humidity control. These procedures resulted in the successful formation of FTO/SnO_2/SSSO films.

3.1 Reaction during annealing temperature.

In this chemical synthesis procedure. When strontium nitrate $Sr(NO_3)_2$ is dissolved in water and deposited on top of tin dioxide SnO_2 and then annealed at 180°C for an hour, there are several possible outcomes, depending on the precise conditions and stoichiometry. However, generally, at this temperature, strontium nitrate could decompose to strontium oxide SrO with the evolution of nitrogen dioxide NO_2 and oxygen O_2. The reaction would be something like this:

$$2Sr(NO_3)_2 \rightarrow 2SrO + 4NO_2 + O_2 \quad \ldots\ldots\ldots\ldots\ldots\ldots..1$$

The strontium oxide could then potentially react with the tin dioxide to form a strontium tin oxide compound, though the exact compound formed would depend on the specific reaction conditions and the ratios of strontium to tin.

$SrO + SnO_2 \rightarrow SrSnO_3$ ……………….2

The XRD spectrum shown in Figure 1 a) (manuscript) reveals the presence of SnO_2, $SrSnO_3$, and SrO under the specified test and reaction conditions. We elucidate the layer structure based on the reaction as follows: SnO_2 acts as the **bottom layer**. The reaction occurring at the top of the SnO_2 surface produces SrO, as in equation 1. Where SrO and SnO_2 meet, they react, forming a new layer of $SrSnO_3$ on top of the SnO_2, which serves as the **middle layer**. According to the XRD test in Figure 1 a) (manuscript), the unreacted SrO forms the **top layer**. Therefore, the final structure is a layer of SnO_2, $SrSnO_3$, and SrO, represented as SnO_2-$SrSnO_3$-SrO (SnO_2-SSSO).

4. Characterization

A. X-ray diffraction test (XRD)

The model of the XRD diffractometer used in this experiment is the German Bruker type. Insert the FTO substrate coated with the sample into the sample tank. Align the surface center of the sample with the axis of the goniometer. The scanning range is 10-50°. The speed is 7°/min.

B. Ultraviolet-visible spectrophotometry test (UV-Vis)

The instrument used for UV-visible spectroscopy in this experiment is the model UV-1800 from Shimadzu Corporation of Japan. Instrument. The liquid sample is placed in a color vessel and tested in the range of 200-800 nm.

C. Field emission scanning electron microscope test (SEM)

This experiment uses the Apero S instrument of the American FEI. The sample cut into a suitable size is fixed on the sample stage with conductive glue. Then a layer of conductive glue is applied

to the surface of the sample to connect it to the metal sample stage, and the surface to be observed is and the cross-section is exposed, and the morphological image is obtained by injecting samples and observing.

D. Optoelectronic performance test

Photoelectric conversion efficiency measurement: The performance of PSCs devices is characterized by the J-V curve. The test system consists of a solar simulator and a digital source meter. The light intensity is AM 1.5 G (100 mW/cm^2), calibrated with a standard silicon photocell before testing. During the test, turn on the light source to preheat, calibrate the light intensity after stabilization, set the scanning parameters (range 1.2-0 V, scanning speed 0.01 V/s, scanning direction is divided into forward and reverse scanning), and the effectiveness of the battery device after adding a light shield the area is 0.04 cm^2. The test collects electrical signals and obtains the J-V curve. Based on the curve, the open circuit voltage (Voc), short circuit current density (Jsc), fill factor (FF), and photoelectric conversion efficiency (PCE) are calculated.

E. X-ray photoelectron spectroscopy test (XPS)

The instrument model used in this experiment is the American Thermoelectric Company's K-Alpha+ instrument, and the radiation source is Al K. The sample is scanned with broad spectrum and narrow spectrum, and the contamination C 1s (284.8 eV) signal position is calibrated.

F. Ultraviolet photoelectron spectroscopy test (UPS)

The instrument model used in this experiment is the AXIS SUPRA multifunctional ray photoelectron spectrometer, which uses an HeI emission line and the vacuum degree is maintained at about <5×10-8 Pa.

G. Photoluminescence spectrum test (PL&TRPL)

The instrument model used in this experiment is FLS 1000. The steady-state PL emission is measured under the excitation of a monochromatized xenon lamp at a wavelength of 470 nm, and the signal detection range is 600-900 nm. Time-resolved fluorescence decay using Measurements are made with a 365nm laser, detecting signals in the 760nm wavelength range.

H. Atomic force microscope test (AFM)

The instrument model used in this experiment is Dimension icon (Bruker Company, Germany), and the test conditions are normal temperature and pressure.

L. Optoelectronic performance test

External quantum efficiency test (EQE): The EQE test system used in this experiment is QE-R type. The test spectrum range is 300-850 nm. It adopts DC test mode with a step size of 10 nm. The instrument used for AC impedance spectroscopy (EIS) testing is the CHI660D electrochemical workstation. The scanning range of the test is 100 mHz to 1 MHz. All tests are performed at an LED AC amplitude of 10 mV. The steady-state output uses a model SS150 solar simulator, calibrating the light intensity through standard silicon, combined with The CHI660D (Shanghai Chenhua) electrochemical workstation was used to measure and track the steady-state output current at the maximum power point by applying a constant bias voltage to the PSCs device. The instrument used to test the current-voltage curve under dark state conditions is the CHI660D electrochemical workstation, and the external bias voltage is from -3 V to 3 V.

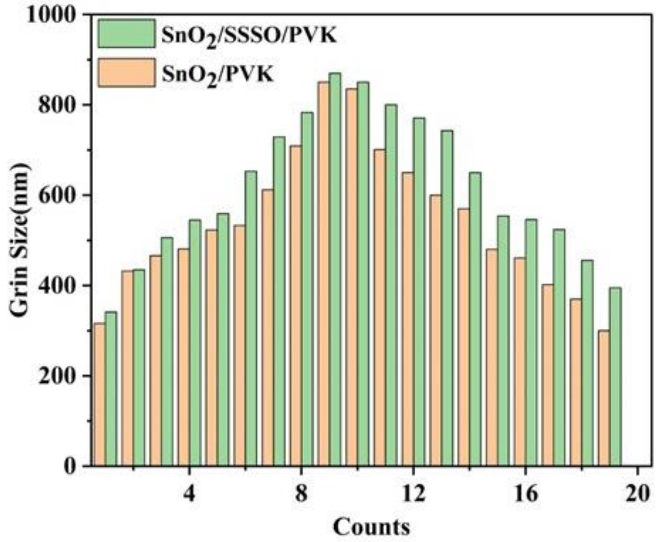

Figure S1 The particle sizes of the perovskite film were measured after the perovskite layer was deposited on substrates of SnO_2 and SnO_2-SSSO.

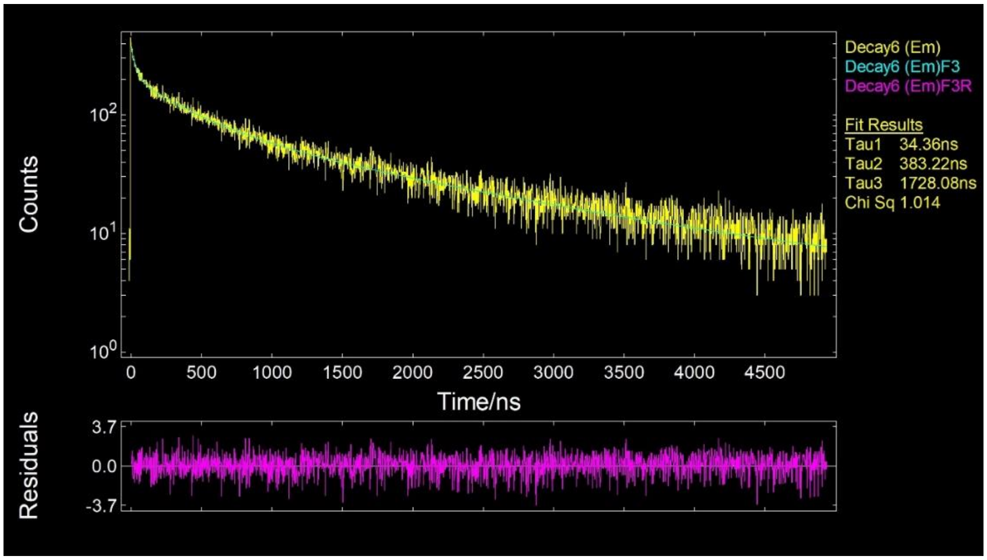

Figure S2 TRPL fitting curve for the SnO_2/perovskite film.

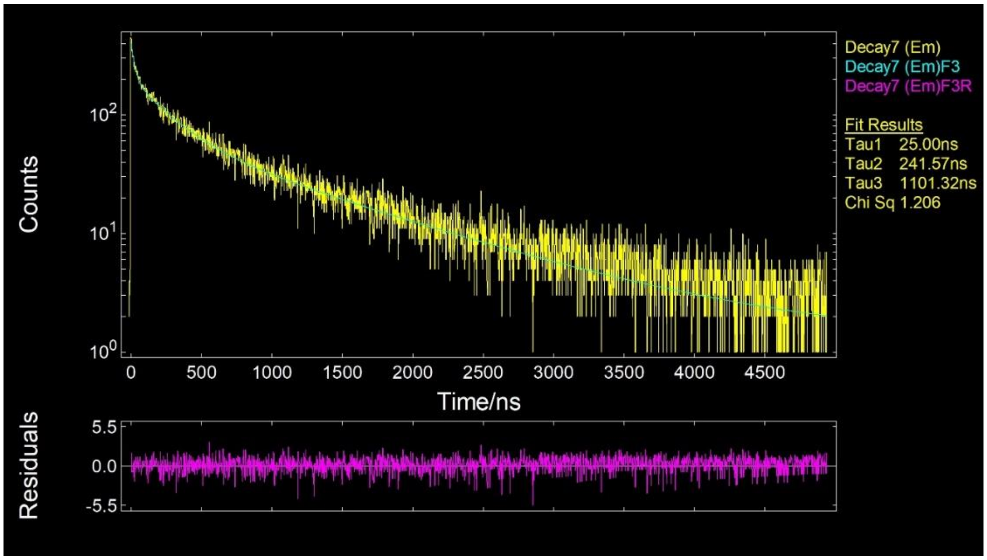

Figure S3 TRPL fitting curve for the SnO_2-SSSO/perovskite film.

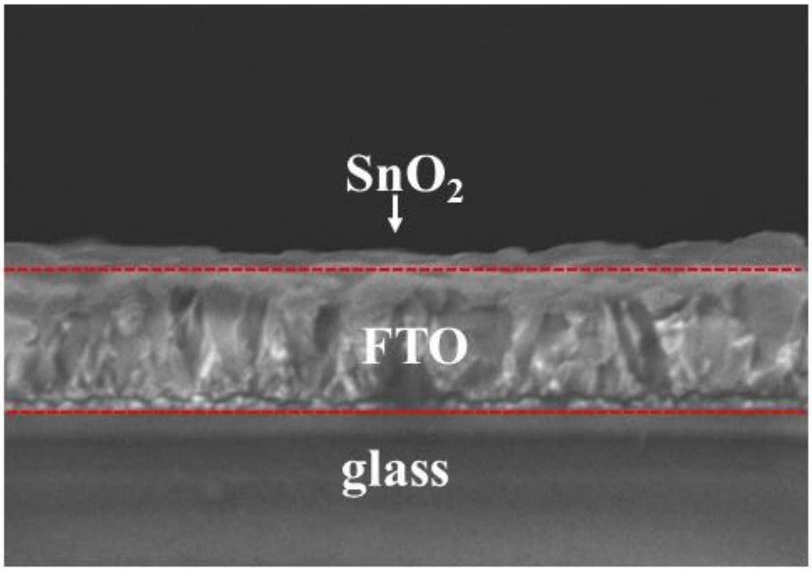

Figure S4 Cross-sectional SEM of the thicker FTO/SnO_2 film.

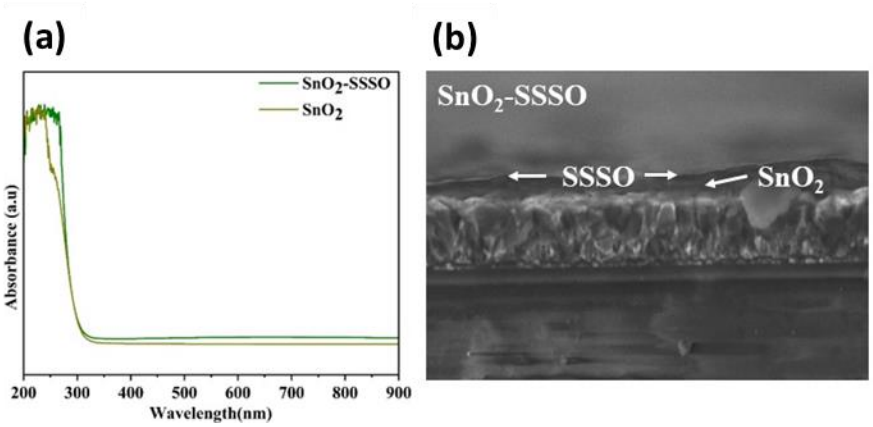

Figure S5 a) UV-Vis of FTO/SnO_2 and FTO/SnO_2-SSSafter and b) before and deposition PVK layer, and c) cross-sectional SEM of FTO/SnO_2-SSSO.

Table S1 TRPL property values for pristine SnO_2 PSCs and treated SnO_2-SSSO PSCs films.

Film	τ_1	B1	τ_2	B2	τ_3	B3	τ_{AVG}
SnO_2-SSSO	25	216.34	241.56	136.78	1101.13	69.83	272.56
SnO_2	34.36	174.51	383.22	123.25	1728.07	81.06	510.30

Table S2 the photovoltaic characteristics of pristine SnO_2 PSC and SnO_2-SSSO PSC devices.

Type	E_{cutoff}	E_{dag}	Φ	Eg	EvB	ECB
SnO_2	17.31	4.03	- 3.91	3.79	- 7.94	- 4.15
SnO_2-SSSO	17.39	4.0	- 3.85	3.77	- 7.83	- 4.06

Table S3 Photovoltaic parameters of devices in both reverse and forward scan directions using SnO_2 and SnO_2-SSSO films.

Type	R_s	C	R_{rec}
SnO_2-SSSO	138.4	6.964E-9	865780
SnO_2	227.2	4.929E-9	815890

Table S4 The fitting parameters obtained from the EIS data of devices based on SnO_2 and SnO_2-SSSO film

Type	PCE(%)	Voc(%)	FF(%)	Jsc(mA/cm^2)	Hysteresis (%)
ReverseSnO_2	21.97	1.12	79.19	24.57	10.92%
Forward SnO_2	19.57	1.12	72.68	24.05	
Reverse SnO_2-SSSO	24.06	1.16	82.66	24.90	4.77%
Forward SnO_2-SSSO	22.91	1.15	80.33	24.81	

Reference

[1] Yoo, J. J., Seo, G., Chua, M. R., Park, T. G., Lu, Y., Rotermund, F & Seo, J. (2021). Efficient perovskite solar cells via improved carrier management. *Nature*, *590*(7847), 587-593.
